# Long-term cognitive outcomes among unselected ventilated and non-ventilated ICU patients

**DOI:** 10.1186/s40560-017-0213-4

**Published:** 2017-02-17

**Authors:** José Raimundo A. de Azevedo, Widlani Sousa Montenegro, Djane Pereira Rodrigues, Suellen C. de C. Souza, Vanessa F. S. Araujo, Margareth Pereira de Paula, Patricia H. C. P. Prazeres, Adenilde da Luz Leitão, Adriana V. N. Mendonça

**Affiliations:** Intensive Care Unit, Hospital São Domingos, Av. Jerônimo de Albuquerque, 540, Bequimao, São Luis, MA 65060-645 Brazil

**Keywords:** Critical illness, Intensive care unit, Follow-up, Cognitive dysfunction, Delirium, Outcome

## Abstract

**Background:**

Cognitive dysfunction is an important long-term complication of critical illness associated with reduced quality of life, increase in healthcare costs, and institutionalization. Delirium, an acute form of brain dysfunction that is common during critical illness has been shown to be associated with long-term cognitive dysfunction. The aim of this prospective cohort study was to estimate the prevalence and severity of cognitive dysfunction in an unselected population of medical and surgical ICU patients.

**Methods:**

This prospective observational cohort study included all adult patients admitted to the surgical (13 beds) and medical (32 beds) ICUs of a tertiary hospital over a 12-month period. Patients with impaired cognition were excluded. At least 3 months after hospital discharge, patients were assessed for cognition using a validated battery of tests and were classified as having no cognitive impairment, mild to moderate cognitive impairment, or severe cognitive impairment.

**Results:**

Four hundred thirteen patients were tested an average of 11 (3–18) months after discharge. Fifty-five (13.3%) patients included in the follow-up cohort had delirium. Cognitive impairment was identified in 206 (49.9%) patients, 120 (29.1%) patients had mild or moderate cognitive impairment, and 86 (20%) patients had severe cognitive dysfunction.

**Conclusions:**

This investigation in an unselected and lower severity population of critically ill patients demonstrates that cognitive dysfunction is a frequent and severe long-term complication.

## Background

The large number of patients being treated annually in intensive care units (ICUs) and the improved care of these patients has resulted in a growing number of critical illness survivors, many of who are left with significant morbidities [1–3]. An important long-term complication of critical illness is cognitive dysfunction, which is associated with a reduced quality of life and an increase in healthcare costs and institutionalization [4, 5]. Delirium, an acute form of brain dysfunction that is common during critical illnesses, has been shown to be associated with long-term cognitive dysfunction [4, 6].

Currently, cognitive dysfunction associated with critical illness is inserted in a broader syndrome context of post intensive care syndrome (PICS), which includes psychiatric and physical dysfunction. Although exact data is not available, it is estimated that at least half of patients discharged from ICUs have at least one manifestation of PICS [7, 8]. In a study of 293 patients that survived ICU admission, 44% required assistance by their community nurse, and a negative impact on family income was reported by one third to one half of patients and families of survivors [9].

The aim of this prospective cohort study was to estimate the prevalence and severity of cognitive dysfunction in an unselected population of medical and surgical patients and to evaluate if delirium duration was an independent determinant of the severity of cognitive dysfunction.

## Methods

This prospective observational cohort study was approved by the Research Ethics Committee of Federal University of Maranhao-Brazil-under number 990.167. Included in the study were all adult patients admitted to the surgical (13 beds) and medical (32 beds) ICUs of a tertiary hospital over a 12-month period (from March 2014 to February 2015). We excluded patients with preexisting cognitive dysfunction due to neurodegenerative disease or central nervous system traumatic or vascular disease; patients admitted to the ICU after cardiac arrest with suspected anoxic brain injury; patients for whom follow-up would be difficult due to active substance abuse, psychotic disorder, or residence outside Sao Luis Island (827 km^2^, five counties); patients who could not be reliably assessed for delirium owing to blindness, deafness or language deficit, and patients for whom informed consent could not be obtained. Patients determined by the psychologist on ICU admission to have evidence of a preexisting cognitive dysfunction were also excluded.

Written informed consent was obtained from the patient or next of kin.

Patients underwent two daily evaluations for delirium (morning and evening) with the use of the Confusion Assessment Method for the ICU (CAM-ICU), a diagnostic tool for determining the presence of delirium based on four features: acute changes or fluctuation in mental status, inattention, disorganized thinking, and altered level of consciousness [10]. Level of consciousness was evaluated with the use of the Richmond Agitation-Sedation Scale (RASS). Scores range from −5 (unarousable) to +4 (agitated) [11]. The duration of delirium was defined as the number of days in which the patient had at least one of the two daily evaluations of CAM-ICU positive for delirium during the ICU length of stay.

At least 3 months after hospital discharge, patients were assessed for cognition using a validated battery of tests including: (1) the forward and backward digit span to assess attention and memory [12]; (2) the Rey Auditory Verbal Learning Test to assess verbal memory [13]; (3) the clock-drawing test to evaluate executive functions [14]; (4) the verbal fluency test to assess language [15]; and the Mini-Mental State Examination to assess global mental status [16]. All these tests have been validated for the Brazilian population and were applied by trained nurses and psychologists and interpreted by a neuropsychologist (MPP). Each patient’s cognitive test scores were converted to *T* scores using age-specific and education-specific normative data. We classified patients as having mild or moderate impairment if they had either two cognitive test scores 1.5 standard deviation (SD) below the mean or one cognitive test score 2 SD below the mean; we classified patients as having severe cognitive impairment if they had three or more cognitive test scores 1.5 SD below the mean or two or more cognitive test scores 2 SD below the mean.

### Statistical analysis

Demographic and clinical characteristics of the in-hospital cohort and follow-up cohort were examined using median and interquartile range and proportions for categorical variables. To determine whether the duration of delirium was an independent predictor of long-term cognitive impairment, we used multiple non-linear regression analysis to analyze the association between days of delirium and summary scores of cognitive performance at follow-up, adjusting for the following covariates: age, education, APACHE IV score, and sepsis.

## Results

From March 2014 to February 2015, 724 patients were enrolled in the clinical trial; 53 patients died during hospitalization and 4 had other criteria for exclusion (1 had a large stroke before discharge and 3 had cardiac arrest with suspected anoxic brain injury during their ICU stay). The remaining 667 patients were eligible for the cohort. Fifty-four (8.0%) patients died before follow-up testing. One hundred forty-five (21.7%) were lost for follow-up (55 of them lived outside the São Luis Island) and 55 (8.1%) patients refused to undergo the cognition assessment. The remaining 413 patients were tested an average of 11 (3–18) months after discharge. Table 1 shows that demographic and clinical data were comparable between the in-hospital and the follow-up group.

**Table 1:** Demographic and clinical characteristics of the patients

	In-hospital cohort (*n* = 724)	Follow-up cohort (*n* = 413)
Age, year		
Median	59	57
IQR	47–73	46–72
Male sex		
*n* (%)	374 (51.7)	206 (50.1)
Education, year		
Median	11	11
IQR	11–14	11–14
APACHE IV score		
Median	35	32
IQR	23–53	21–48
SOFA score at		
Enrollment		
Median	1	1
IQR	0–3	1–3
Mechanical ventilation		
No. of patients (%)	92 (12.3%)	51 (13.4%)
No. of days		
Median	4	3
IQR	2–10	1.2–6.7
Use of sedative agent		
No. of patients (%)	88 (12.1%)	44 (10.7%)
Diagnosis at admission		
*n* (%)		
AMI, CHF, Arrhytmia	146 (20.2)	98 (23.7)
Acute respiratory failure^a^	109 (15.1)	58 (14.0)
Other surgical procedures^b^	99 (13.7)	56 (13.6)
Neurologic disease	93 (12.8)	53 (12.8)
Sepsis, septic shock	77 (10.6)	37 (9.0)
Digestive surgery	69 (9.6)	37 (9.0)
Digestive disease	67 (9.3)	39 (9.4)
Other diagnosis	64 (8.7)	35 (8.5)
Delirium		
No. of patients (%)	80 (11.6)	55 (13.3)
No. of days		
Median	4	3
IQR	2–5	2–5
Duration at ICU stay		
Median	11	10
IQR	6–23	5–19

^a^Acute respiratory failure included acute respiratory distress syndrome, pneumonia, acute exacerbation of chronic pulmonary disease, asthma, pulmonary edema, and embolism

^b^Other surgical procedures included orthopedic, vascular, and urologic surgery

Ninety-two patients (12.3%) of the in-hospital cohort were submitted to mechanical ventilation for a median of 4 days (interquartile range, 2–10) compared to 51 (13.4%) with a median duration of 3 days (interquartile range, 1.2–6.7) in the follow-up cohort. Sepsis and septic shock were the admission diagnosis in 77 (10.6%) of the patients of in-hospital cohort and 37 (9.0%) of the follow-up cohort patients. Two hundred sixty-nine (65.2%) of the evaluations were done in the patients’ homes. One hundred forty-four patients (34.8%) were evaluated in the psychology follow-up clinic. Fifty-five (13.3%) patients included in the follow-up cohort had delirium. Cognitive impairment was identified in 206 (49.9%) patients; 120 (29.1%) had mild or moderate cognitive impairment and 86 (20%) severe cognitive dysfunction (Table 2).

**Table 2:** Cognitive outcomes during follow-up

	Follow-up assessment (*n* = 413)
No impairment	207 (50.1)
Mild/moderate	120 (29.1)
Impairment	
Severe impairment	86 (20.8)

**Fig. 1:**
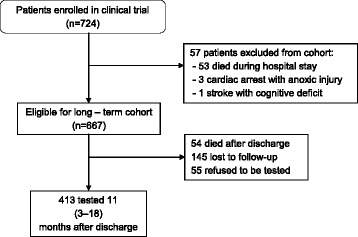
Enrollment and follow-up

Eleven (34.3%) patients who had delirium for 3 days or more presented with severe cognitive dysfunction (*p* = 0.17). In logistic regression analysis, a duration of delirium for 3 days or more was not an independent predictor of cognitive dysfunction (*p* = 0.76). In addition to delirium, we evaluated other factors that could affect post intensive care cognitive dysfunction: gender, ICU LOS, hospital LOS, years of education, APACHE IV score, SOFA score at enrollment, and Charlson comorbidity index (CCI). In univariate analysis variables SOFA score, years of education, and CCI were significant (Fig. 1). When evaluated in the multivariate logistic regression analysis only CCI had a borderline significance (*p* = 0.06).

## Discussion

For a long time, the success of therapeutic interventions in critically ill patients was evaluated by the in-hospital mortality rate. However, it is becoming increasingly evident that hospital discharge as an endpoint needs to be reevaluated. Critically ill patients often develop a series of long-term complications during and after their hospital stay, including cognitive impairment, psychological disorders, and muscle weakness. These changes can persist for years and seriously affect the quality of life [5, 9–19]. Prolonged cognitive dysfunction is one of the most prominent manifestations of PICS [7, 8].

We studied a very different group of patients than what has been investigated before [4, 6, 18, 19]. Here, we sought not to study the sickest of the sick ICU patients, but on the contrary, we chose this unselected and most lower severity of illness group to determine in this group what the rates of long-term cognitive impairment are. It is surprising and alarming that we have found such high rates of a wide spectrum of cognitive impairment in a group of patients whom most ICU clinicians would never suspect this degree of long-term cognitive impairment. Furthermore, since this group was of such a lower severity of illness, it was not alarming that they experienced most less acute brain dysfunction. In fact, with this low prevalence of delirium [only about 1 in 10 in comparison to upwards of 7 out of 10 in sicker ICU cohorts [4]], it is not surprising that our analyses did not see a relationship between delirium and long-term cognitive impairment, though this analysis is severely under-powered and thus may represent a type II error. This should not be taken by the readership to be a statement that delirium (acute brain dysfunction in the ICU) is not a risk factor for long-term cognitive impairment but rather that our investigation was simply unable to assess this risk factor. However, this makes it even more interesting that we still found such a high amount of long-term cognitive impairment, which raises the specter that other risk factors besides delirium must also be considered and should be investigated. As nearly 1 in 3 of our patients had either cardiac or neurological injury, both of which can pose major immediate problems to the brain in terms of perfusion, this is a very important consideration especially since this is the major difference between who we enrolled and studied versus previous cohorts looking at long-term cognitive impairment in ICU survivors [6, 18, 19].

This study reinforces the findings of previous studies that showed a high prevalence of cognitive dysfunction in critically ill patients [1, 3–6]. More important, however, was the finding of a 50% incidence of cognitive impairment (20% severe), in an unselected low severity of illness group of clinical and surgical patients. Most other studies evaluated specific nosologies such as acute respiratory distress syndrome, older adults, and patients admitted to medical ICU [1, 17–19]. The studies of Girard et al. [6] and Pandariphande et al. [4] which demonstrated a close relationship between cognitive dysfunction and delirium analyzed only patients undergoing mechanical ventilation [6] and patients with respiratory failure and cardiogenic and septic shock [4].

One strength of this study is that in spite of being a cohort the in-hospital population did not differ significantly from the follow-up population in terms of age, years of formal education, and severity, incidence, and duration of delirium, reducing the possibility that patients who were sicker were underrepresented. This is in contrast to a large, multicenter, prospective cohort study that exhibited clear differences between the patients that completed the neurocognitive testing and those that did not [4].

Our study has limitations. The single-center design limits the generalization of results to other similar populations. Admissions to the ICU are not elective in most cases. Thus, evaluation of the presence of pre-existing cognitive dysfunction has limitations. Although we excluded patients determined by a psychologist on ICU admission to have evidence of preexisting cognitive dysfunction, it is possible, nevertheless, that patients with mild cognitive dysfunction were included in the study.

## Conclusions

To our knowledge, this prospective observational cohort study is the first investigation in an unselected and lower severity population of critically ill medical and surgical patients to demonstrate that cognitive dysfunction is a frequent and severe long-term complication in survivors of critical illness.

The results of this study reinforce the evidence that many patients who survive critical illness evolve after discharge with a chronic condition characterized by cognitive dysfunction and also muscle dysfunction and psychiatric disorders, the post intensive care syndrome (PICS). Prevention of PICS begins during the patient’s stay in the ICU and needs to continue after discharge with a multidisciplinary approach involving the patient and their family.
